# Expectations About Precision Bias Metacognition and Awareness

**DOI:** 10.1037/xge0001371

**Published:** 2023-03-27

**Authors:** Helen Olawole-Scott, Daniel Yon

**Affiliations:** 1 Department of Psychological Sciences, Birkbeck, University of London; 2 Department of Psychology, Goldsmiths, University of London

**Keywords:** metacognition, precision, perception, expectation, uncertainty

## Abstract

Bayesian models of the mind suggest that we estimate the reliability or “precision” of incoming sensory signals to guide perceptual inference and to construct feelings of confidence or uncertainty about what we are perceiving. However, accurately estimating precision is likely to be challenging for bounded systems like the brain. One way observers could overcome this challenge is to form *expectations* about the precision of their perceptions and use these to guide metacognition and awareness. Here we test this possibility. Participants made perceptual decisions about visual motion stimuli, while providing confidence ratings (Experiments 1 and 2) or ratings of subjective visibility (Experiment 3). In each experiment, participants acquired probabilistic expectations about the likely strength of upcoming signals. We found these expectations about precision altered metacognition and awareness—with participants feeling more confident and stimuli appearing more vivid when stronger sensory signals were expected, without concomitant changes in objective perceptual performance. Computational modeling revealed that this effect could be well explained by a predictive learning model that infers the precision (strength) of current signals as a weighted combination of incoming evidence and top-down expectation. These results support an influential but untested tenet of Bayesian models of cognition, suggesting that agents do not only “read out” the reliability of information arriving at their senses, but also take into account prior knowledge about how reliable or “precise” different sources of information are likely to be. This reveals that expectations about precision influence how the sensory world appears and how much we trust our senses.

## Public Significance Statement

Thinking about the human mind has been transformed by the idea that the brain is Bayesian—making inferences about the world by tracking the uncertainty or “precision” of different sources of information. Over the past few years, our laboratory has conducted theoretical and empirical research on Bayesian models of perceptual and metacognitive inference. Recently, we have argued that a key unanswered question about modern Bayesian accounts is whether and how agents form expectations about precision (Yon & Frith, 2021). In this article, we test this idea, and find evidence that agents do indeed form expectations about the clarity or “precision” of sensory signals they are likely to encounter.

Bayesian models of the mind suggest that successful perception, action, and cognition depend on estimating uncertainty. For example, tracking the uncertainty of our perceptual systems allows us to engage in sophisticated forms of monitoring and control. Imagine you are driving your car as the sun begins to set. As the sunlight wanes, the information landing at your senses becomes less reliable, leading to less accurate percepts. Importantly, by tracking these changes in sensory reliability, we can act in ways that optimize perception and action. For example, we might turn on the headlights to make the road ahead clearer.

Psychologists and neuroscientists have often thought about this kind of uncertainty monitoring through Bayesian models and the idea of *precision*. Bayesian accounts assume that agents track the uncertainty of their own internal states by tracking the noise or variability in different parts of their cognitive machinery. For example, Bayesian models of perception suggest that observers estimate the precision of incoming evidence and use these estimates to decide how to combine information from different sensory systems (Ernst & Banks, 2002) or how to combine incoming evidence and prior expectations (Yon & Frith, 2021)—giving more weight to incoming signals that are currently most precise. In a similar vein, Bayesian models of metacognition have suggested that explicit feelings of confidence about what we are perceiving are generated by reading out the uncertainty or precision in sensory circuits—such that we are more confident when our sensory representations are less noisy (Geurts et al., 2022).

However, an important shift in contemporary Bayesian models is the idea that precision is not estimated based on incoming evidence alone. Recent accounts also assume that agents form probabilistic beliefs about how precise information is *likely* to be, and these expectations are incorporated into precision estimates (Friston, 2018). Allowing precision to decouple from momentary reality in this way has allowed researchers to develop myriad explanations for diverse aspects of experience and awareness. For example, Bayesian theories of psychosis have suggested that inappropriately strong beliefs about the imprecision of sensory signals (relative to expectations) could lead to unusual and distressing experiences like hallucinations (Corlett et al., 2019).

**Figure 1 fig1:**
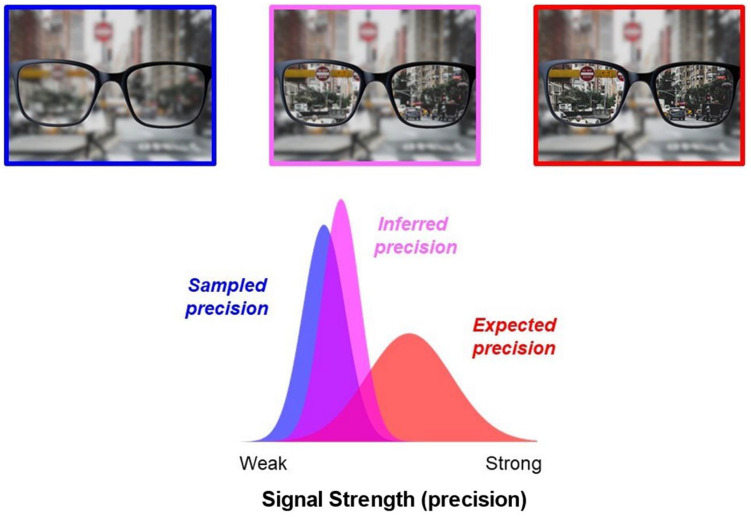
Expectations Bias Precision Estimation *Note*. Contemporary Bayesian models suggest that we estimate the precision of our senses by combining incoming evidence with prior expectations about how reliable signals are likely to be. This is usually a good idea but could lead to biases when expectation and reality diverge. For example, if we pick up the wrong pair of glasses, we may expect our vision to improve (red) but the actual signals sampled by vision may remain noisy and imprecise (blue). If we combine this expectation and evidence, we may thus erroneously infer that our vision is more reliable than it truly is (pink). See the online article for the color version of this figure.

Forming beliefs about precision would help agents to estimate uncertainty—which may often be difficult to compute (Yon & Frith, 2021). Combining incoming evidence with *expectations* about precision based on past experience could optimize metacognitive monitoring of perception. For example, we may *expect* based on past experience that putting on our glasses will improve the fidelity of incoming visual signals. Since this expectation often comes true, incorporating this prior knowledge into our beliefs will improve our estimates of perceptual precision. However, while combining expectations and incoming signals to estimate precision will usually be adaptive, such a process will also lead to errors when expectation and reality diverge (see Figure 1). For example, if we mistakenly leave the house with an old pair of glasses that have the wrong prescription, we may believe that putting on our glasses will improve perceptual precision more than it truly does—leading us to overconfidence in our perceptual abilities, with potentially serious consequences as we take our car for a spin.[Fig fig1]

This idea of *expected precision* has become increasingly embedded in theoretical models of the Bayesian brain. For example, recent models of hierarchical predictive coding suggest that our brain also entertains a kind of “shadow hierarchy” alongside the primary information streams—with separate neural populations encoding beliefs about the precision of ascending evidence and descending predictions at different hierarchical levels (Friston, 2018). Indeed, computational models based on these frameworks have relied on the concept of expected precision to explain perception (Kanai et al., 2015) and to model false perceptual inferences (Parr et al., 2018). However, while we can potentially explain various aspects of perception and metacognition by assuming agents form beliefs about precision, we do not currently know how or whether expectations about perceptual precision are actually formed. There is extant evidence that agents can predict their decision confidence in a variety of settings (e.g., Boldt et al., 2019; Daniel & Pollmann, 2012; Fleming et al., 2016; Guggenmos et al., 2016), but this does not necessarily entail that agents track or form predictions about the reliability or precision of incoming signals. Without evidence that precision is indeed learned and inferred, it may be premature to use this framework to explain diverse aspects of cognition in health and disease.

The present study addresses this gap. Here we investigate whether agents form beliefs about the reliability of incoming sensory signals, and whether these beliefs influence perceptual metacognition in the way that contemporary Bayesian models propose. Participants completed a perceptual decision-making task, judging the direction of moving dots. Crucially, probabilistic cues manipulated expectations about signal strength across trials, such that observers could expect motion clouds to be *strong* or *weak*. To pre-empt our results, across three experiments we found that these expectations biased perceptual confidence (Experiments 1 and 2) and subjective visibility ratings (Experiment 3) such that observers were more confident, and stimuli appeared more vivid, when stronger signals were expected. We find this bias in subjective awareness can be captured by a computational model which assumes that agents form expectations about the signals they are likely to encounter in different contexts and infer the strength (precision) of sensory signals by combining these expectations and incoming evidence from the senses.

Taken together, these results support the idea that we estimate the precision of our senses by combining current sensory evidence with expectations about how precise this evidence is likely to be. This provides support for a widespread but untested tenet of influential Bayesian models of metacognition, revealing that expectations about precision influence how the sensory world appears and how much we trust our senses.

## Experiment 1

Experiment 1 tested whether participants form expectations about the reliability of incoming signals, and how these beliefs influence perceptual metacognition. Participants completed a perceptual decision-making task, judging whether clouds of dots moved left or right. Importantly, probabilistic cues signaled whether sensory signals would likely be strong or weak. We probed how expectations established by these cues biased perceptual confidence.

### Method

#### Participants

Thirty-four participants (21 female, 13 male, *M*_age_ = 33.9 years, *SD* = 8.45) were recruited via Prolific. All participants reported normal or corrected vision and no history of psychiatric or neurological illness. This sample size was selected to provide 80% power to detect at least a medium-sized effect (Cohen's dz = 0.5). This value was not explicitly guided by prior work (unlike Experiments 2 and 3). All experiments were approved by the Research Ethics Committee at Goldsmiths, University of London.

Participants who failed to complete at least 90% of trials across the training and test phase were excluded. Participants were considered outliers if their individual effects (i.e., condition-wise differences in accuracy or confidence) were >2.5 *SD*s away from the sample mean. We identified outliers based on participant's condition-wise effects (rather than specific condition means or trial-level data). Outliers were winsorized to values 2.5 *SD*s away from the mean for inferential statistics, rather than adjusting raw datapoints. The same was true for all experiments. For Experiment 1, no participants were excluded and only one condition-wise effect for confidence scores was winsorized. Data patterns and their statistical significance were unchanged by this adjustment.

#### Procedure

**Figure 2 fig2:**
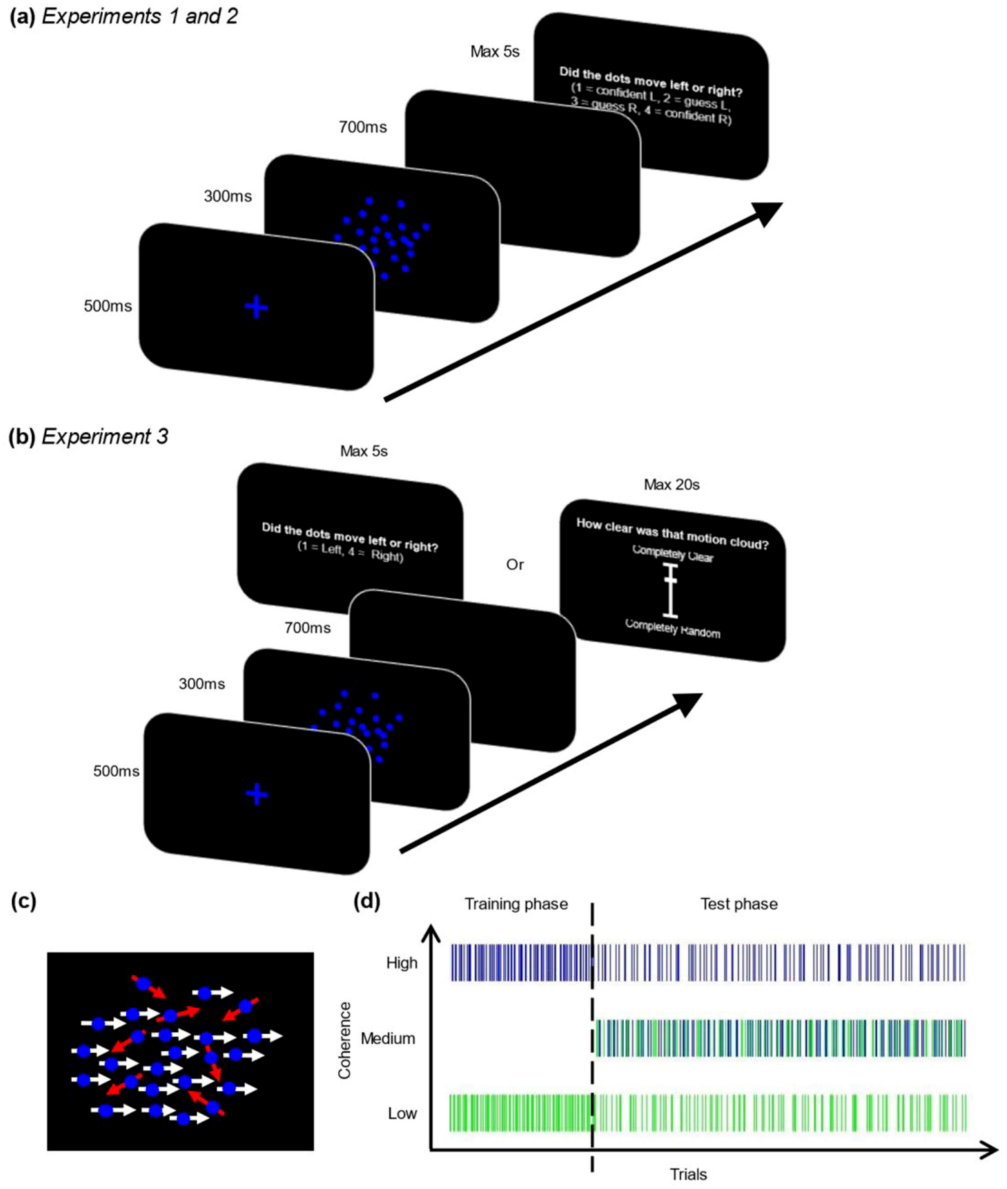
Experimental Task *Note*. (a) Participants completed a motion perception task, judging the direction of brief motion clouds and reporting confidence in their decision. Color cues manipulated expectations about the strength of motion patterns for each trial, for example, if stimuli were blue participants could expect high motion coherence. (b) On *medium probe trials* in Experiment 3, the perceptual decision was replaced by a visibility scale. (c) Illustration of “motion coherence”: in each stimulus, a proportion of dots was programmed to move left or right (white arrows) while the remainder of dots moved in random directions (red arrows). Manipulating the proportion of coherent dots changes the strength of the motion signal. (d) Example timecourse of trials across the experiment: The training phase consisted of perfectly deterministic mappings between color and coherence. During the test phase, half of the trials were identical to those shown during training, whereas the other half paired the same color cues with objective perceptual signals of medium strength. See the online article for the color version of this figure.

Participants completed an online perceptual decision-making task programmed in PsychoPy (Peirce et al., 2019), discriminating patterns of moving dots and reporting confidence in their perceptual choices (see Figure 2). Each trial began with a fixation cross (500 ms) before the presentation of a dot motion stimulus (300 ms). In each motion cloud, a proportion of dots was programmed to move coherently left or coherently right, while the remaining dots moved in random directions. After a blank screen (700 ms) participants gave a combined report of their perceptual decision (left or right) and confidence level (confident or guess) on a 4-point scale.[Fig fig2]

Critically, probabilistic cues (colors) allowed observers to form expectations about the strength of motion signals on each trial, allowing us to investigate how such expectations bias perceptual confidence. For example, for a given observer when the fixation cross and stimulus dots appeared in green, motion clouds were likely to have low coherence (i.e., 4% motion coherence—weak signals; see Figure 2). In contrast, when stimuli appeared in blue, motion clouds were likely to have high coherence (i.e., 52% motion coherence—strong signals). Color mappings were counterbalanced across participants and participants were not explicitly informed about the association between the probabilistic cues and signal strength.

The experiment comprised 560 trials (see Figure 2). The first 160 trials acted as an initial training phase to establish expectations about color cues. Here participants experienced perfectly deterministic mappings between color and coherence, for example, every blue stimulus was programmed to be a strong signal, and every green stimulus was programmed to be a weak signal.

Participants then completed a 400-trial test phase. Half of these trials were identical to what participants experienced during training, where the color cues were followed by the predicted signal strength (e.g., the color cue associated with strong signals was followed by objectively strong motion). However, our key trials of interest in this phase were the remaining half of *medium probe* trials. On these trials, participants received the same color cues but received an objective perceptual signal of medium strength (28% motion coherence). Given objective signal strength is identical on these trials, any differences in objective accuracy or subjective confidence on these trials must reflect the effects of expectations about signal strength.

### Results

We investigated how actual and expected precision altered perception and metacognition by computing measures of objective and subjective performance from perceptual choices and confidence ratings. Objective perceptual sensitivity was measured by calculating the proportion of correct decisions (accuracy) and *d*′. We also looked at reaction times on these trials, as a measure of performance. To capture subjective aspects of metacognition, we calculated “confidence level,” that is, the proportion of decisions participants report with high rather than low confidence. We also calculated meta-*d*′ and *M*_ratio_ as complementary measures of metacognitive sensitivity and efficiency—measuring how closely subjective confidence ratings (“confident” or “guess”) track decision accuracy, and whether this changes while controlling for differences in task performance (Maniscalco & Lau, 2012). We computed *d*′, meta-*d*′, and *M*_ratio_ using the nonhierarchical variant of the HMeta-d toolbox (Fleming, 2017). Inferential tests used an alpha level of .05, and nonsignificant results were qualified with equivalent Bayesian analyses. These yielded Bayes factors (BF) that quantified evidence for an effect (H_1_) over evidence for the null (H_0_). Conventionally, BF_10_ < 0.33 denotes moderate evidence in support of a null effect.

First, we compared perception and metacognition on trials where motion signals were objectively stronger or weaker. Unsurprisingly, perceptual decisions were more accurate when signals were objectively stronger (*M*_accuracy_ = 0.939, mean *d*′ = 3.672) than when they were objectively weaker (*M*_accuracy_ = 0.564, mean *d*′ = 0.349; accuracy: *t*_33_ = 21.606, *p* < .001, dz = 3.705; *d*′: *t*_33_ = 18.061, *p* < .001, dz = 3.097). Participants also reported higher confidence in perceptual decisions when objective signal strength was strong (*M* = 0.843) compared to when signals were objectively weak (*M* = 0.341, *t*_33_ = 11.200, *p* < .001, dz = 1.921).

**Figure 3 fig3:**
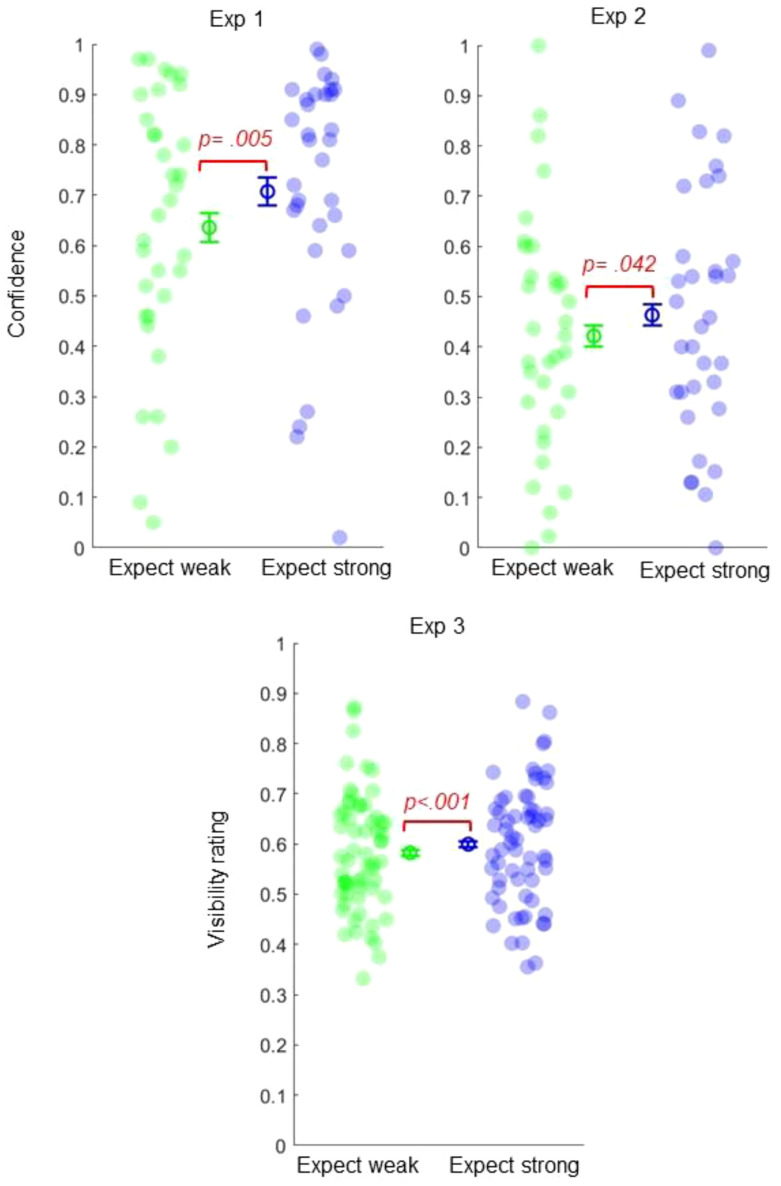
Expected Precision Alters Metacognition and Awareness *Note*. Participants reported significantly higher confidence (Experiments 1 and 2) and higher visibility ratings (Experiment 3) on “expect strong” trials. Error bars represent 95% within-subject confidence intervals. See the online article for the color version of this figure.

However, our key question concerns how *expected* precision alters perception and metacognition. This can be evaluated by comparing our test trials where participants *expect* strong or weak signals—but receive objectively identical medium coherence stimuli. These comparisons revealed that participants were more confident in their decisions on *expect strong* trials—*M* (*SEM*) = 0.71 (0.042)—than *expect weak* trials—*M* (*SEM*) = 0.64 (0.045), *t*_33_ = 3.015, *p* = .005, dz = 0.517 (see Figure 3). Exploratory analyses revealed that this confidence bias did not differ between correct and incorrect perceptual decisions (see the online supplemental materials).[Fig fig3]

**Figure 4 fig4:**
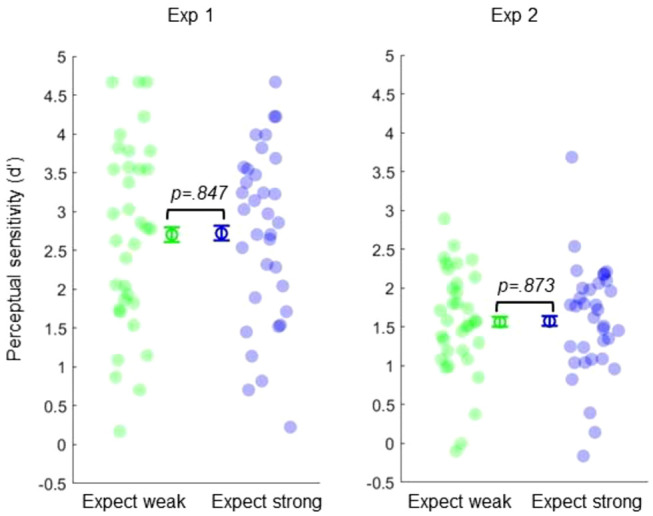
Expected Precision Does Not Alter Objective Sensitivity *Note*. Perceptual sensitivity (*d*′) was unaffected by probabilistic cues. Error bars represent 95% within-subject confidence intervals. See the online article for the color version of this figure.

Importantly, this difference in confidence arose even though objective perceptual accuracy and *d*′ scores did not significantly differ between these conditions (accuracy: *t*_33_ = 0.913, *p* = .368, dz = 0.156, BF_10_ = 0.270; *d*′: *t*_33_ = 0.194, *p* = .847, dz = 0.033, BF_10_ = 0.187, respectively; see Figure 4). There was also no difference in reaction time (*t*_33_ = 1.257, *p* = .217, dz = 0.216). There was also no significant difference in meta-*d*′, nor *M*_ratio_, between conditions (meta-*d*′: *t*_33_ = 1.544, *p* = .132, dz = 0.265, BF_10_ = 0.539; *M*_ratio_: *t*_33_ = 0.146, *p* = .884, dz = 0.025, BF_10_ = 0.186)—suggesting that expectations about precision induce a metacognitive bias, rather than altering the discriminability of introspective states.[Fig fig4]

### Discussion

Experiment 1 suggests that expectations about signal strength bias perceptual metacognition, such that perceptual confidence is exaggerated when participants expect more precise (i.e., high coherence) motion signals—even if such strong signals do not actually ensue. This is consistent with Bayesian theories that suggest we form expectations about the precision of sensory signals (Friston, 2018), which in turn shape beliefs about the reliability of the senses (Yon & Frith, 2021).

## Experiment 2

Experiment 1 found evidence consistent with the Bayesian idea that expectations about signal strength bias perceptual metacognition. This inference was based on the fact that perceptual confidence differed for objectively identical stimuli according to whether the observer *expected* a strong or weak signal, while perceptual and metacognitive sensitivity remained unchanged.

In Experiment 1, these medium “test” stimuli were chosen as the midpoint of coherence (28%) between weak (4%) and strong (52%) signals participants experienced throughout the task. While this makes the medium test stimuli the objective intermediate point between the signals, in Experiment 1, such stimuli were found to not be intermediate in terms of decision difficulty. In particular, accuracy on medium test trials (*M* = 0.872) was more similar to decision accuracy with strong (*M* = 0.939) rather than weak signals (*M* = 0.564).

This discrepancy is potentially important for understanding the underlying mechanism at play in Experiment 1. For example, it is possible that expecting strong signals actually improves the *sensitivity* of metacognition, such that participants are better able to detect their correct decisions, rather than directly inducing a confidence bias (as has been suggested in prior work—Sherman & Seth, 2021). When accuracy is near ceiling, an improvement in metacognitive sensitivity may appear to induce a bias in overall confidence—since accurate insight will lead to higher confidence ratings.

This alternative explanation seems unlikely given that Experiment 1 found expectations biased perceptual confidence but did not significantly alter metacognitive sensitivity (meta-*d*). However, to ensure the reliability of these effects and to rule out this alternative explanation we ran Experiment 2. Experiment 2 was a preregistered replication of Experiment 1 with one key change: the coherence of *medium probe* trials was lowered to ensure participants would no longer approach ceiling on decision accuracy. If expectations about precision directly bias confidence, Experiment 2 should replicate the findings of Experiment 1—finding expectations about signal precision induce a bias in confidence ratings but leave metacognitive sensitivity unaffected.

### Method

#### Participants

For Experiment 2, 34 new participants (15 female, 19 male, *M*_age_ = 37.3, *SD* = 9.28) were recruited via Prolific. This sample size was selected to provide 80% power to detect effects at least as large as those found in Experiment 1 (effect of expectation on confidence level—dz = 0.517). The same exclusion and outlier identification procedures were used as in Experiment 1, and the experiment was preregistered on AsPredicted (https://aspredicted.org/bs8ww.pdf). No participants were excluded, and winsorization was applied to one participant's condition-wise effect in the confidence level comparison—though this adjustment did not affect data patterns or their significance.

#### Procedure and Paradigm

Experiment 2 used the same procedure and paradigm as Experiment 1, except the coherence level of the middle signal strength trials was decreased from 28% to 16% motion coherence.

#### Preregistered Hypotheses and Analyses

We preregistered the prediction that confidence ratings would be higher when participants expected strong sensory signals (https://aspredicted.org/bs8ww.pdf). We preregistered that we would also analyze accuracy, *d*′, and meta-*d*′ but did not preregister any predictions that these would differ between conditions. Analyses of *M*_ratio_ and reaction times were suggested by anonymous reviewers.

### Results

The same measures from Experiment 1 (accuracy, *d*′, reaction times, confidence, meta-*d*′ and *M*_ratio_) and statistical analyses were also employed for Experiment 2.

Experiment 2 replicated the results of Experiment 1. Again, participants were more accurate when signals were objectively stronger (mean accuracy= 0.945, mean *d*′ = 3.761) than when they were objectively weaker (mean accuracy= 0.567, mean *d*′ = 0.339; accuracy: *t*_33_ = 20.444, *p* < .001, dz = 3.506; *d*′: *t*_33_ = 18.177, *p* < .001, dz = 3.117). Participants also reported higher confidence in perceptual decisions when objective signal strength was strong (*M* = 0.800) compared to when signals were weak (*M* = 0.294, *t*_33_ = 10.332, *p* < .001, dz = 1.772).

More importantly, participants reported higher confidence on *expect strong* trials—*M* (*SEM*) = 0.46 (0.042) compared to *expect weak* trials—*M* (*SEM*) = 0.42 (0.041), *t*_33_ = 2.114, *p* = .042, dz = 0.362 (see Figure 3). Exploratory analyses again revealed that this bias did not differ between correct and incorrect perceptual decisions (see the online supplemental materials).

Again, these differences in confidence were obtained even though there was no significant difference in accuracy (*t*_33_ = 0.118, *p* = .907, dz = 0.020, BF_10_ = 0.185) or *d*′ between conditions (*t*_33_ = 0.161, *p* = .873, dz = 0.028, BF_10_ = 0.186; see Figure 4). There are also no differences in reaction times (*t*_33_ = 1.146, *p* = .260, dz = 0.196). Critically, while this change in confidence level was replicated, expectations about signal precision had no significant effect on metacognitive sensitivity (meta-*d*)—*t*_33_ = 0.261, *p* = .796, dz = 0.045, BF_10_ = 0.190, nor metacognitive efficiency (*M*_ratio_)—*t*_33_ = 0.998, *p* = .326, dz = 0.171, BF_10_ = 0.291.

### Discussion

Experiment 2 replicated the results of Experiment 1—again finding that observers are biased to feel more confident in perceptual choices when stronger signals are expected. These effects are consistent with Bayesian models that assume agents form beliefs about the precision of incoming signals and use these expectations to guide perceptual metacognition. Importantly, Experiment 2 also rules out the possibility that these effects arise because of changes in metacognitive sensitivity rather than bias.

However, this is not the only interpretation. An alternative possibility is that this bias in confidence arises because agents form expectations about their *performance* in different contexts, rather than expectations about the precision of evidence per se. For example, previous work has found that agents readily form beliefs about the difficulty of different tasks even in the absence of explicit feedback—and can use these global performance estimates to guide decisions about which goals to pursue (Rouault et al., 2019). Indeed, recent results suggest that these kinds of expectations about confidence and task difficulty may also directly bias prospective and retrospective decision confidence (Boldt et al., 2019; Van Marcke et al., 2022). Under this alternative way of thinking, effects in our experiments may be generated by metacognitive mechanisms which track the fact that decisions tend to be more accurate in one color context than another. Learning about the probability of being correct could also bias decision confidence (Fleming & Daw, 2017), even if agents are not learning or forming expectations about the clarity or precision of incoming signals—but simply learn that they feel more confident in some contexts rather than others, without learning why.

To evaluate this alternative possibility, we ran Experiment 3—testing more directly whether observers acquire expectations about precision, and whether these expectations shape inferences observers make about the strength and clarity of incoming sensory signals.

## Experiment 3

Experiments 1 and 2 found that participants reported higher confidence in perceptual decisions when expecting strong signals. Such biases could be driven by changes in apparent signal strength—such that when the participant is expecting a stronger signal, they overestimate the precision of incoming sensory information, leading to exaggerated feelings of confidence. However, as noted above, this effect could also reflect participants forming a confidence bias that is unrelated to signal precision—for example, learning that decisions tend to be easier in the blue context rather than the green context, without tracking signal strength to learn this.

Experiment 3 was conducted to determine whether learning in our paradigm biases the apparent reliability of perceptual signals, rather than inducing a generic confidence bias. To this end, we replaced confidence ratings with a more direct assay of apparent signal strength—the subjective clarity of the visual motion.

### Method

#### Participants

For Experiment 3, 62 new participants (36 female, 26 male, *M*_age_ = 35.4, *SD* = 7.52) were recruited via Prolific, using the same selection criteria as Experiments 1 and 2. The sample size was chosen to provide 80% power to detect effects at least as large as those seen on confidence level in Experiment 2 (Cohen's dz = 0.362). The experiment used the same exclusion and outlier identification criteria as Experiments 1 and 2 and was preregistered on AsPredicted (https://aspredicted.org/zk64a.pdf). No participants were excluded. Winsorization was applied to one participant's condition-wise effect (in the visibility rating comparison), but this did not alter statistical patterns or their significance.

#### Procedure and Paradigm

Participants completed the same task used in Experiment 1 with two critical changes. The metacognitive report was removed entirely, such that participants only reported motion direction (left or right) and never rated decision confidence. On the critical *medium probe* trials in the test phase, participants did not make judgments about motion direction. Instead, a visibility scale appeared post-stimulus, asking participants to judge “how clear was that motion cloud?” on a continuous scale ranging from “completely random” to “completely clear” (see Figure 2). Ratings from this scale were used as an index of subjective awareness, providing an assay of how clear (or “precise”) visual signals appeared. Importantly, removing the perceptual decision on these trials means that participants must make an estimate about the sampled signal, rather than estimating the correctness of an explicit choice.

The overall structure of the experiment remained the same as Experiments 1 and 2, except the number of training phase trials was increased to 200. During the test phase, the visibility scale was displayed on all *medium probe* trials instead of the perceptual decision. To prevent participants from learning that the visibility scale was only presented on trials where the objective signal strength was truly intermediate, the scale was also presented on 10% of the high and low coherence trials in the test phase.

#### Preregistered Hypotheses and Analyses

We preregistered the prediction that visibility ratings would be biased by participant's expectations about signal strength (https://aspredicted.org/zk64a.pdf).

### Results

As in Experiments 1 and 2, perceptual decisions were more accurate when signals were objectively stronger (mean accuracy = 0.972, mean *d*′ = 3.911) compared to when they were objectively weaker (mean accuracy = 0.562, mean *d*′ = 0.326; accuracy: *t*_61_ = 55.718, *p* < .001, dz = 7.076; *d*′: *t*_61_ = 41.220, *p* < .001, dz = 5.235). Participants also reported higher subjective visibility ratings when objective signal strength was strong (*M* = 0.74) compared to when signals were weak (*M* = 0.417, *t*_61_ = 15.236, *p* < .001, dz = 1.935).

Critically, analyses also revealed that expectations about precision altered visibility ratings, even when objective signal strength was matched. Observers reported that medium strength stimuli appeared more vivid when stronger signals were expected—*M* (*SEM*) = 0.6 (0.015)—compared to when signals were expected to be weak—*M* (*SEM*) = 0.582 (0.015), *t*_62_ = 3.673, *p* < .001, dz = 0.467 (see Figure 3).

### Discussion

It was unclear from Experiments 1 and 2 whether our learning manipulation causes participants to form expectations about signal strength, or expectations about performance confidence. In Experiment 3, participants rated the subjective clarity of motion, rather than reporting decision confidence. Here we found that observers were biased to rate identical motion clouds as seeming clearer when more precise signals were expected. This is consistent with the possibility that our learning manipulation causes observers to form expectations about perceptual precision which in turn alter how strong signals appear to be. These changes in apparent signal quality can also plausibly explain why decision confidence is higher when stronger signals are expected.

## Computational Modeling

Experiment 3 found that stimuli appeared more vivid when the observers expected stronger signals. Such an effect could arise if subjective vividness reflects an inference of signal strength, which observers form by combining the bottom-up sensory evidence with top-down predictions about how strong or “precise” sensory evidence is likely to be in a given context (Friston, 2018; Yon & Frith, 2021). We used computational modeling to evaluate this possibility.

In our model, agents learn and make inferences about the signals they encounter in different contexts throughout the task. On each trial, observers receive a stimulus with a certain signal strength—ranging from completely random motion to completely coherent motion in one direction. As a first step, we assume the model has access to these stimulus energies trial-by-trial. We estimated the motion energy in each stimulus by calculating the horizontal motion component of each moving dot (given by the cosine of the motion angle). Averaging these motion components across all dots in the display yields an estimate of signal strength bounded between 1 (all dots move coherently in one direction) and 0 (no motion signal at all). While the motion signal present on any given trial is strongly determined by its programmed coherence (i.e., higher coherence clouds tend to have stronger signals), there can still be substantial variability between motion clouds with the same programmed coherence—depending on how the random dots in the cloud behave.

Our model assumes that agents use these samples of motion energy to learn expectations about the likely signal strengths in the two cue contexts (i.e., blue context and green context), which in turn shape estimates about signal strength on a given trial. The model implements this by assuming that an inference of signal strength (*inference*) on trial *t* is produced by computing a weighted average of the sampled sensory evidence (*evidence*) and prior expectation (*prior*), where *w*_prior_ and *w*_evidence_ are the respective weights applied to expectations and evidence in this combination:

$$\text{inferenc}{\text{e}}_{t}\;=\;w_{\mathrm{prior}}(\text{prio}{\text{r}}_{t})+\;w_{\mathrm{evidence}}(\text{evidenc}{\text{e}}_{t})$$

$$w_{\mathrm{evidence}}=\;1-\;w_{\mathrm{prior}}$$

In this equation, there is only one free parameter—*w*_prior_ —which controls the relative impact that prior expectation and current evidence have on internal estimates of signal strength. If *w*_prior_ = 1, the observer's current belief about signal strength is entirely determined by their previous experience in this context, ignoring the present stimulus entirely. In contrast, if *w*_prior_ = 0 internal beliefs about signal strength are entirely driven by the quality of the current stimulus and past experience is discarded.

Importantly, the model iteratively combines learning and inference, such that once a belief about signal strength has been formed on trial *t*, this becomes the new prior for that context on trial *t* + 1. This is analogous to iterative Bayesian updating schemes where prior and evidence are combined at one timepoint to form a posterior, which becomes the new prior for the next timepoint, and so on. Importantly, this means that *w*_prior_ also effectively acts as a learning rate parameter. For values of *w*_prior_ closer to 0, expectations for trial *t* + 1 are driven mostly by signals experienced on trial *t*. In contrast, for values of *w*_prior_ closer to 1, predictions are more strongly driven by the accumulation of past experiences rather than current evidence. This dual role for the parameter *w*_prior_ in inference and learning is reminiscent of hierarchical Bayesian models of message passing in the brain, which assume a common parameter simultaneously determines how strongly prior knowledge is weighted when making inferences and how stubborn these prior hypotheses are in the face of new data (Friston, 2018; Yon et al., 2019). Indeed, under certain assumptions, this learning model can be shown to be equivalent to models of Bayesian inference, where the combination of prior and evidence is controlled by the (estimated) precision of each information source (see Supplementary Modeling in the online supplemental materials).

**Figure 5 fig5:**
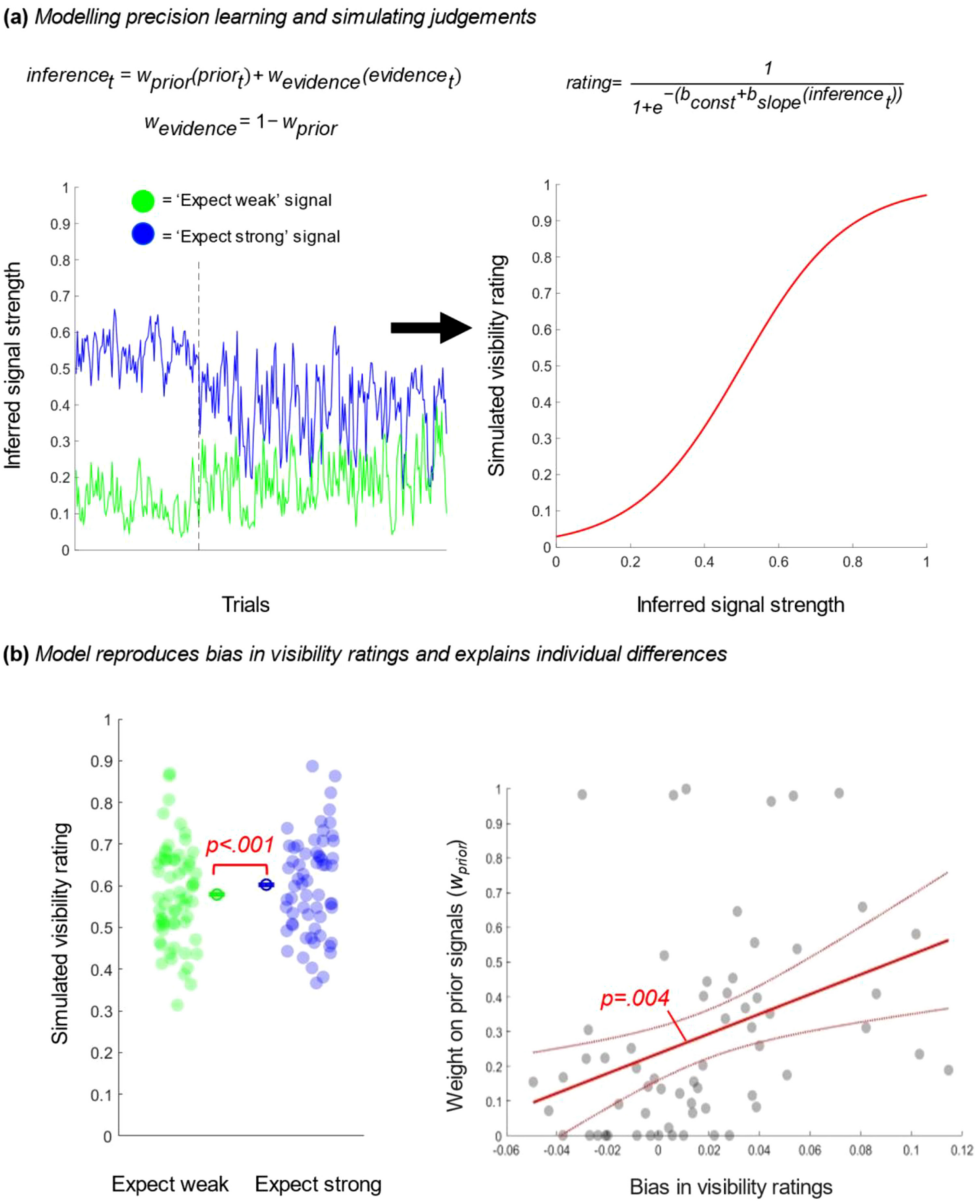
Modeling Precision Learning *Note*. (a) Our model assumes that observers form inferences about signal strength by computing a weighted combination of incoming evidence and past experiences. This generates a trajectory of beliefs about signal strength across trials (left). Our model assumes when observers are probed to rate the visibility of a stimulus, they pass this momentary belief through a mapping function to generate a rating (right). Dashed line in left panel denotes transition from training phase to test phase. (b) Analyzing simulated data in the same way as real data found that the model reproduced the key result of Experiment 3—the model rates stimuli as being more visible when stronger signals should be expected (left). There was a strong correlation between the model weight on prior experience (*w*_prior_) and the empirical bias observed for each participant (higher values = stronger bias). Red lines display best linear fit and confidence bounds. See the online article for the color version of this figure.

This process yields a trajectory of beliefs about signal strength that integrates past experience and current evidence—controlled by the parameter *w*_prior_ (see Figure 5).[Fig fig5]

The final step in the model turns trial-wise beliefs about signal strength into ratings on the visibility scale. This is achieved by taking the internal inference of signal strength on a given trial and passing this through a logistic function of the form:

$$\text{rating}=\;\frac{1}{1+e^{-(b_{\mathrm{const}}+b_{\mathrm{slope}}(\mathrm{inferenc}e_{t}))}}$$

This transfer function in the model reflects our assumption that agents form beliefs about signal strength that they communicate in potentially noisy or biased ways. This accords with ideas from metacognition research (Bang et al., 2020; Guggenmos, 2022) and reinforcement learning (Lockwood & Klein-Flügge, 2021) where decisions and actions reflect a noisy transfer of an internal belief into an overt choice. This function produces a continuous rating of motion vividness bounded between 0 (completely random) and 1 (completely clear), controlled by two parameters—*b*_slope_ and *b*_const_. *b*_slope_ determines the gradient of the function mapping internal estimates of signal strength to visibility ratings—such that higher values indicate a tight mapping between beliefs and ratings, and lower values indicate a noisier translation from inferences to ratings (*b*—beta). The *b*_const_ parameter is a constant value that captures idiosyncratic biases to give high or low visibility ratings irrespective of current inferences about signal strength. (N.B. Model comparison suggested that our data were best fit by a model with this transfer function, rather than by an alternative model where visibility ratings reflect an untransformed readout of inferred signal strength. See Supplementary Modeling in the online supplemental materials.)

To investigate whether this three-parameter model could capture empirical patterns seen in Experiment 3, for each participant we simulated belief trajectories for values of *w*_prior_ ranging between 0 and 0.999 and subsequently found values of *b*_slope_ and *b*_const_ that best predicted the empirical visibility ratings participants gave on medium strength test trials. Maximum likelihood estimation of the logistic transfer function allowed us to identify the combination of best-fitting parameters that minimized the deviance between model and data (i.e., maximized model evidence).

Identifying the best-fitting parameters for each participant allows us to simulate how the model behaves in the experiment, and to investigate whether the model reproduces the observed empirical effects. Analyzing simulated data in the same way as real data found that the model reproduced the key result of Experiment 3—yielding higher subjective visibility ratings on medium test trials when stronger rather than weaker signals were expected (*t*_61_ = 7.366, *p* < .001, dz = 0.935; see Figure 5). Moreover, we found a strong correlation between the size of the empirical effect for each participant and the size of this effect predicted by the model (*r* = .731, *p* < .001).

Analyzing parameter values allowed us to examine which aspects of the model contribute to its ability to reproduce these empirical effects. We found a strong relationship between values of parameter *w*_prior_ and the empirical effect observed for each participant in Experiment 3—*r* = .363, *p* = .004—suggesting that those participants who showed the largest effects of expectations about signal strength were those the model estimated to be placing the greatest weight on prior knowledge (see Figure 5).

## General Discussion

Influential theories suggest that the mind is *Bayesian*—computing the uncertainty or precision of internal representations to guide perception, action, and cognition. In particular, Bayesian accounts of metacognition propose that we build representations of perceptual confidence by estimating the precision of representations in our sensory systems (Geurts et al., 2022). However, an important recent shift in Bayesian frameworks has been the emerging idea that precision estimates are not simply “read out” from sensory systems but formed by combining incoming evidence with learned expectations about how reliable sensory evidence is likely to be. This idea has been and continues to be very influential across the cognitive sciences but has not been directly tested (Yon & Frith, 2021). Here we tested this possibility by manipulating participant's expectations about precision (signal strength) and measuring how these altered perceptual metacognition and subjective awareness.

Our results support the idea that agents combine incoming evidence with learned expectations to estimate the precision of sensory information. We found that participants reported higher confidence (Experiments 1 and 2) and more vivid percepts (Experiment 3) when they expected signals to be stronger—even though objective signal strength was identical, and objective perceptual performance remained unchanged. These results were complemented by computational modeling, which revealed such biases could be well explained by assuming agents infer the precision of sensory signals by combining immediate evidence from their perceptual systems with expectations about how strong signals are likely to be (Friston, 2018; Yon & Frith, 2021).

These results and modeling provide support for an untested tenet of contemporary Bayesian brain models—which suggest that inferences about the precision of our senses incorporate expectations about precision. However, our learning model is not itself Bayesian. Indeed, our model is more closely related to learning algorithms like the Rescorla–Wagner rule (or delta rule), which assumes that agents form and update beliefs by using point estimates (Rescorla & Wagner, 1972). However, under some assumptions, this model becomes mathematically equivalent to Bayesian models of precision-weighted inference, where the combination of incoming evidence and prior expectation is controlled by the relative of uncertainty in each estimate (see Supplementary Modeling in the online supplemental materials). Though this makes it possible to conceptualize our model in Bayesian terms, in the present work we do not have any evidence that the combination of incoming evidence and prior expectations when estimating precision is itself controlled by uncertainty in the evidence or the prior. Future work directly measuring or manipulating uncertainty in evidence or expectations will be important for determining whether we should conceptualize this kind of learning in fully Bayesian terms, rather than the simpler mechanics of our point estimate model (see Supplementary Modeling in the online supplemental materials for full discussion). However, even if future work finds that inferences of precision are not themselves precision-weighted, the present findings still provide support for a conjecture at the heart of modern Bayesian models of the mind—that estimates of precision are shaped by what we expect.

These results have important implications for our understanding of metacognition and perceptual monitoring. One influential conceptualization defines metacognitive states as those that represent uncertainty in our overt and covert decisions (Pouget et al., 2016). This distinguishes metacognition from other kinds of metarepresentation in the mind and brain (Shea, 2014). In this way of thinking, perceptual precision estimates are meta-representational, because they represent uncertainty about the perceptual world. But under this definition, they are not strictly “metacognitive,” as they do not directly represent uncertainty in our decisions.

However, even if perceptual precision estimates are not metacognitive in this sense, they can still support important perceptual monitoring functions—allowing observers to estimate the clarity of their senses. Critically, these estimates of perceptual evidence strength can then form an important component of strictly metacognitive computations like decision confidence (Mamassian, 2016). Though the computations underlying confidence are more complex than a simple “read out” of evidence strength (Aitchison et al., 2015; Fleming & Daw, 2017; Meyniel et al., 2015; Sanders et al., 2016), many models also assume that biases to over- or under-estimate the strength of sensory evidence should also translate into biases in decision confidence—as we have found in the present work.

For example, normative models propose that we estimate the confidence in our perceptual decisions using estimates of the uncertainty in our perceptual circuits (Geurts et al., 2022). However, it is likely to be difficult for systems like the brain to monitor uncertainty based on incoming signals alone (Yon & Frith, 2021). Our results suggest metacognitive mechanisms may finesse this problem by incorporating prior knowledge into these computations—inferring how reliable our senses are by combining current evidence from our perceptual systems with expectations about how precise they are likely to be (Friston, 2018). This will often be adaptive because expectations about precision will often come true. For example, I may *expect* my vision to improve when I put on my glasses, and if I have the right prescription; this expectation is valid. However, relying on expectations may lead to false metacognitive inferences when prediction and reality do not coincide. For example, if I have picked up the wrong pair of glasses, I may expect to see more clearly but actually be more myopic than before. In these cases, relying too heavily on expected precision will lead to overconfidence and maladaptive action based on unreliable evidence.

Our findings demonstrate that agents form expectations about sensory precision which directly alter inferences about signal quality. Previous models (Fleming & Daw, 2017) and experiments (Rouault et al., 2019; Sherman & Seth, 2021) have assumed that agents form metacognitive expectations about task performance—often conceptualized as expecting a high or low probability of being correct. Such ideas gel with computational accounts of metacognition, which define metacognitive processes as exclusively being those involved in computing the probability that a decision is correct (Pouget et al., 2016). Here, we find expected precision biases perceptual confidence (Experiments 1 and 2) but also see these expectations directly alter judgments of signal strength even when no decision is required (Experiment 3). These results may suggest an intermediate stage of perceptual monitoring between lower level perception and higher level metacognition, where agents compute the strength of incoming signals rather than the accuracy of their decisions per se. Indeed, elegant neuroimaging work has found neural representations encoding the quality or vividness of sensory signals that are distinct from those encoding decision confidence (Bang & Fleming, 2018; Mazor et al., 2022). The results we report here are thus compatible with a view where expected precision alters these mid-level representations of signal quality (Yon & Frith, 2021): directly altering how reliable signals appear to observers, which in turn biases later computations of decision confidence that depend on this information.

Experiment 3 investigated whether agents genuinely form expectations about signal strength, rather than performance confidence alone. This was achieved by asking participants to rate subjective clarity of motion clouds rather than reporting decision confidence. Results showed that observers were biased to rate identical motion clouds as seeming clearer when more precise signals were expected—even when this was probed independently of any “decision.” Conceptually, it makes sense to distinguish visibility ratings from confidence ratings, since one judgment asks about properties of the stimulus and the other asks about properties of the decision maker (and indeed, the two kinds of ratings often empirically decouple—Davidson et al., 2022; Rausch & Zehetleitner, 2016; Skewes et al., 2021). However, it remains possible in principle that covert metacognitive processes may influence visibility ratings—such that a participant judges a stimulus is more visible because they judge they could (hypothetically) make an accurate decision about it if probed. Future work could assess this possibility by creating paradigms where confidence and visibility are more strongly decorrelated. This could be achieved by altering the base rates of stimuli to create conditions where participants are highly confident in their judgments about low visibility targets (see Sherman et al., 2015), or by varying decision boundaries orthogonally to stimulus strength (Bang & Fleming, 2018).

Another important question for future work is whether the changes in metacognition and awareness identified here have consequences for metacognitive control. Researchers typically define metacognitive monitoring mechanisms as those processes involved in tracking information in low-level systems for representation at the meta-level (Nelson & Narens, 1990). A paradigmatic example of such monitoring is decision confidence. The principal reason this kind of monitoring has adaptive value, though, is that these estimates of uncertainty can be fed to metacognitive control mechanisms that drive adaptive behaviors to improve cognition and performance (Boldt et al., 2019; Boldt & Gilbert, 2022). This includes slowing down decisions (Yeung & Summerfield, 2012), manipulating our environment (Risko & Gilbert, 2016), seeking information (Desender et al., 2018), or asking for advice when we are uncertain (Bahrami et al., 2010; Shea et al., 2014). Here, we see that expectations about precision alter metacognitive monitoring—ultimately biasing how confident people feel about their percepts. It is important for future work to establish whether these expectation-induced biases in awareness and confidence also influence this kind of metacognitive control.

Our findings demonstrate that expectations shape precision estimates used at high levels of our cognitive architecture. We find these expectations alter confidence and awareness. However, Bayesian models suggest that precision estimation is important for diverse cognitive functions—including perception, learning, and social cognition (Yon & Frith, 2021). One possibility is that our cognitive system maintains a single representation of perceptual precision which is used to support all of these functions. However, we have suggested recently that different precision estimates are maintained at different levels of the hierarchy—and that expectations may exert a stronger influence on precision at higher levels (Yon & Frith, 2021). It is unclear from these findings whether expected precision will also change low-level perceptual inferences. It will thus be important for future work to establish whether expectations about precision exert a similar role on precision-weighted inferences in other domains.

For example, Bayesian models of multisensory integration suggest that observers combine signals from different modalities according to their estimated precision, lending more weight to sensory channels that have the least noise (Alais & Burr, 2004; Ernst & Banks, 2002). Similarly, Bayesian models of prediction suggest that observers make perceptual inferences by combining incoming evidence with probabilistic expectations—leaning more on prior knowledge when the evidence is more ambiguous, that is, less precise (Olkkonen et al., 2014; Press et al., 2020; Yon, 2021). It is possible that the precision representations used to solve these combination problems are also shaped by expectations. For example, observers may learn to expect that their vision is unreliable in some contexts and use this *expectation* to control whether they rely on other senses or other kinds of knowledge when trying to make sense of the world around them. However, it also remains possible that these precision estimates are not shaped by expectations—and that low-level processes like perception use precision estimates that are more closely tied to the objective uncertainty of incoming signals rather than prior beliefs. Understanding whether and how expectations alter precision estimates at different levels of the cognitive hierarchy will constrain theorizing about Bayesian models of the mind—clarifying when and whether beliefs about uncertainty detach from reality (Yon & Frith, 2021).

Understanding these constraints will be particularly important when using the idea of precision to explain unusual experiences and atypical cognition. For example, a prominent explanation of hallucinations in psychosis suggests that these unusual experiences arise because patients hold inappropriate beliefs about the relative precision of incoming sensory signals and top-down predictions, leading to a disproportionately strong weight on prior expectations when perceiving the world (Corlett et al., 2019). These accounts depend on the idea that beliefs about precision can be false, and this could arise if observers hold the wrong *expectations* about precision. However, for this account to be plausible, it would also have to be true that expectations about precision do indeed control processes like perceptual inference. More generally, theories that explain atypical cognition by appealing to atypical precision weights rely on the assumption that precision weights can indeed be learned or adjusted—which remains to be tested across domains (Yon & Frith, 2021).

Nonetheless, here we have seen evidence that expectations about precision can influence metacognition, and atypicalities in this process could plausibly underwrite certain psychiatric symptoms. For example, patients with psychosis frequently hold delusional beliefs that are resistant to revision in the face of new evidence (Davies et al., 2018; Stuke et al., 2018). This resistance could arise through atypicalities in the learning and prediction mechanisms we describe here—as forming inappropriate expectations about the (un)reliability of incoming data from the external world could lead to recalcitrant beliefs that are difficult to update (Yon et al., 2019).

The current study provides strong support for influential Bayesian models of cognition, showing that agents combine incoming evidence with prior expectations to estimate the precision of their senses. These results begin to reveal the mechanisms we use to learn about uncertainty in our own minds and reveal that expectations about precision formed through such learning exert an influence on how we experience the sensory world and how much we trust our senses.

## Supplementary Material

10.1037/xge0001371.supp
