# Supplementary material for: Expectations About Precision Bias Metacognition and Awareness
Source: J Exp Psychol Gen. 2023 Mar 27;152(8):2177–89. doi: 10.1037/xge0001371 (PMC10399087; doi:10.1037/xge0001371)
Supplement: Supplementary file 1 [file xge0001371.docx]

**Expectations about precision bias metacognition and awareness**

Helen Olawole-Scott and Daniel Yon

**Supplementary Material**

***Supplementary Results***

1. **Assessing the effect of accuracy on the identified confidence bias**

For Experiments 1 and 2, participants identified whether patterns of dots moved to the left or right while also reporting confidence in their perceptual choices. We conducted exploratory analyses to assess whether expectations about precision (induced by probabilistic cues), biased confidence equally for correct and incorrect perceptual judgements. This involved analysing confidence ratings with repeated measures ANOVAs with ‘Expectation’ (Expect Strong, Expect Weak) and ‘Accuracy’ (Correct, Incorrect) as factors.

For both Experiments 1 and 2, there was a significant main effect of Accuracy – with participants reporting higher confidence on correct trials than incorrect trials (Exp1: F_(1,33)_= 324.726, p< .001, η_p_^2^= 0.908; Exp2: F_(1,33)_= 81.198, p< .001, η_p_^2^= 0.711). These analyses also revealed significant main effects of Expectation – where observers rated higher confidence when expecting stronger signals – (Exp1: F_(1,33)_= 10.419, p= .003, η_p_^2^= 0.240; Exp2: F_(1,33)_= 5.273, p= .028, η_p_^2^= 0.138) – which equivalent to the pair-wise effect tested with the t-tests in the main manuscript.

Critically however there was no significant interaction between these two factors in either Experiment 1 or 2 (Exp1: F_(1,33)_= 1.456, p= .236, η_p_^2^= 0.042; Exp2: F_(1,33)_= 0.001, p= 0.971, η_p_^2^= 4.176^e-05^). This suggests that whether the participant was incorrect or correct in the initial perceptual judgement (i.e., identifying whether the dots were moving left or right) did not modulate the subsequent bias in confidence caused by expectations.

1. **Comparing alternative measures of motion signal strength**

The current manuscript quantifies the overall signal strength of dot motion stimuli by averaging the cosine of each dot’s angle of trajectory. We explored whether this motion energy estimation procedure generated similar results to other procedures, such as the motion energy model developed by Adelson and Bergen (1985).

To this end, we simulated 1000 possible dot motion stimuli (like those used in our experiment) and computed motion energy estimates using our calculation and the Adelson & Bergen method. For the latter, we used a MATLAB implementation of the Adelson & Bergen model developed by George Mather (http://www.georgemather.com/Model.html). As shown below, there was a very close correspondence between these estimates – r = 0.911, p <.001.


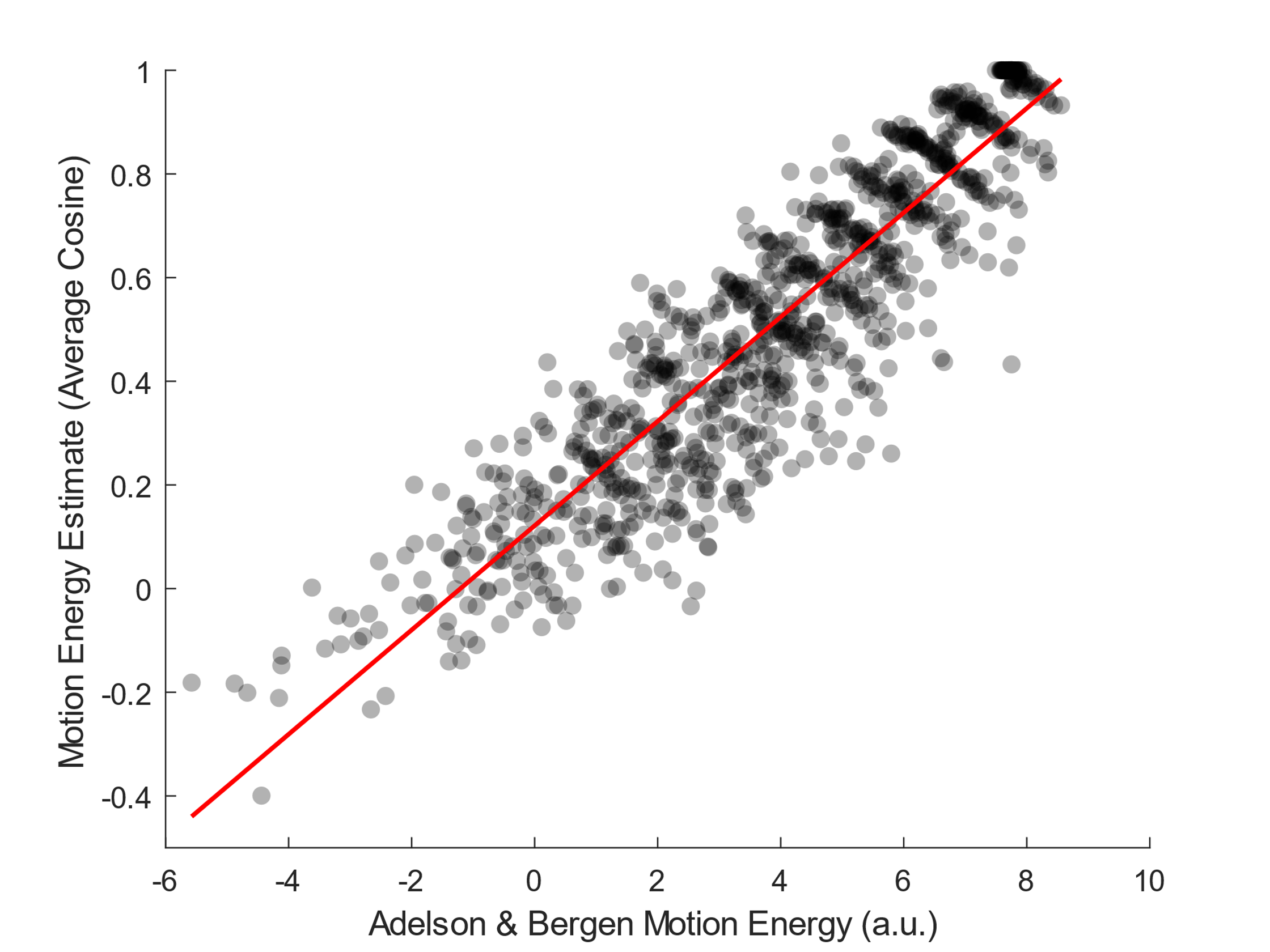


Supplementary Figure 1 – Correlation between current method of quantifying signal strength (mean of angle cosines) with Adelson & Bergen’s model of motion energy.

1. **Visualisations of within-subjects variability**


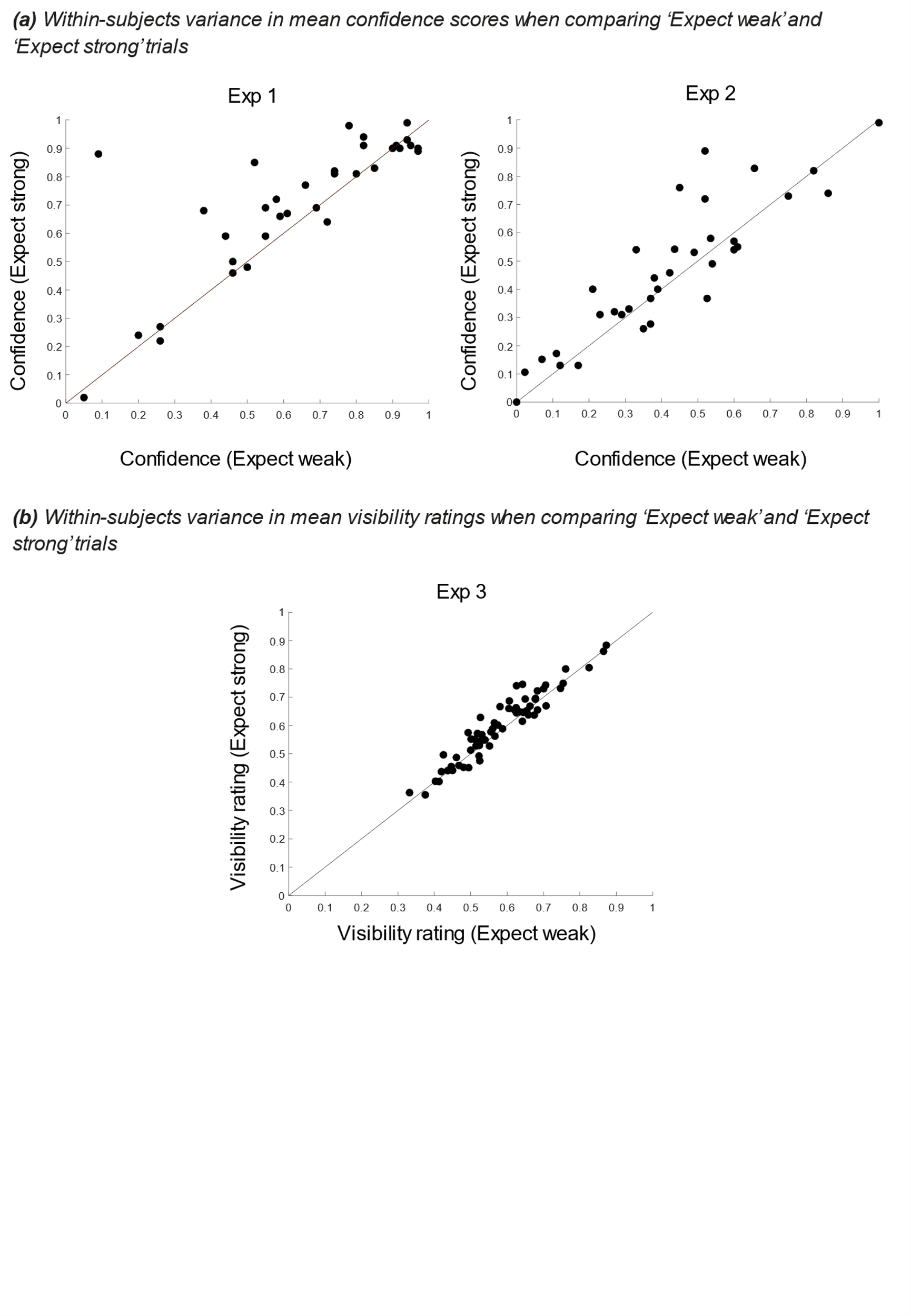


Supplementary Figure 2 - (a) mean confidence ratings and (b) mean visibility ratings for ‘Expect weak’ vs. ‘Expect strong’ medium coherence trials to illustrate within-subject variability.

***Supplementary modelling***

1. **Weighted combination of point estimates**

The model we present in this paper assumes that agents form an estimate (or inference) of the signal strength at timepoint *t* as a weighted combination of incoming evidence and prior expectation:

$\text{inference}_{\text{t}}\text{=}\text{w}_{\text{prior}}\left( \text{prior}_{\text{t}} \right)\text{+ }\text{w}_{\text{evidence}}\text{(}\text{evidence}_{\text{t}}\text{)}$ (1.1)

Where the weights on prior and evidence are defined as:

$\text{w}_{\text{prior}}\text{=}\text{ }\text{1}\text{-}\text{ }\text{w}_{\text{evidence}}$ (1.2)

This model, in itself, makes no particular assumptions about how prior, evidence and inference are represented in the mind and brain – aside from assuming that agents have access to a point estimate of these variables at a given point in time. By trading only in point estimates, this weighted combination is similar to classic models of associative learning such as the Rescorla-Wagner learning rule (also known as the ‘delta rule’; ((Dayan & Kakade, 2000; Rescorla & Wagner, 1972)). Indeed, substituting Eq 1.2 into Eq 1.1 and rearranging yields:

$\text{inference}_{\text{t}}\text{=} \text{prior}_{\text{t}}\text{+}{\text{ }\text{w}}_{\text{evidence}}\text{(}\text{evidence}_{\text{t}} \text{-} \text{prior}_{\text{t}}\text{)}$ (1.3)

This is identical to the Rescorla-Wagner rule, where a point estimate - $\text{inference}_{\text{t}}$ - is made by computing a prediction error - ${\text{(}\text{evidence}}_{\text{t}} \text{-} \text{prior}_{\text{t}}\text{)}$, - and using this difference value to update an initial expectation - $\text{prior}_{\text{t}}$. The size of this update is controlled by $\text{w}_{\text{evidence}}$, which is equivalent to the learning rate parameter in Rescorla-Wagner, usually denoted as *α (alpha)*. Given this equivalence and Eq 1.2, one could also think of $\text{w}_{\text{prior}}$ is equivalent to 1 – *α*.

1. **Relationship to models of Bayesian inference**

However, while the model presented in this paper only assumes that agents form point estimates, it is also possible to connect these ideas to models of Bayesian inference. In particular, we could imagine that agents represent both the incoming evidence and their prior expectation as Gaussian distributions. These two Gaussian distributions are then combined together to form an inferred (posterior) distribution, which is also Gaussian. As shown in the figure below, each of these Gaussians is centred on a mean - *µ (mu) -* while the width of each distribution is controlled by the standard deviation -$\text{σ}$ *(sigma)*.


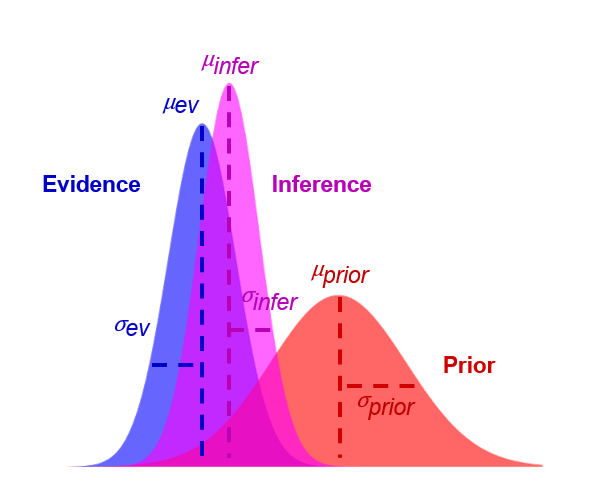


The Bayes-optimal estimate for the inferred (posterior) distribution is given by combining the evidence and expectation distribution according to their precision, where precision is the inverse variance - $\frac{\text{1}}{\text{σ}^{\text{2}}}$. This means that more weight is given to the source of information (evidence or expectation) that is estimated to be most precise. In particular, the Bayes-optimal estimate for $\text{μ}_{\text{infer}}$ is:

$\text{μ}_{\text{infer}}\text{= }\text{w}_{\text{prior}}\left( \text{μ}_{\text{prior}} \right)\text{+ }\text{w}_{\text{ev}}\left( \text{μ}_{\text{ev}} \right)$ (2.1)

Where:

$\text{w}_{\text{prior}}\text{= }\frac{\frac{\text{1}}{{\text{σ}^{\text{2}}}_{\text{prior}}}\text{ }}{\frac{\text{1}}{{\text{σ}^{\text{2}}}_{\text{prior}}}\text{+ }\frac{\text{1}}{{\text{σ}^{\text{2}}}_{\text{ev}}}}$ (2.2)

And:

$\text{w}_{\text{ev}}\text{=} \frac{\frac{\text{1}}{{\text{σ}^{\text{2}}}_{\text{ev}}}\text{ }}{\frac{\text{1}}{{\text{σ}^{\text{2}}}_{\text{prior}}}\text{+} \frac{\text{1}}{{\text{σ}^{\text{2}}}_{\text{ev}}}}$ (2.3)

Because the denominators are the same, these weights sum to 1:

$\text{w}_{\text{prior}}\text{ + }\text{w}_{\text{ev}}\text{= }\frac{\frac{\text{1}}{{\text{σ}^{\text{2}}}_{\text{prior}}}\text{ }}{\frac{\text{1}}{{\text{σ}^{\text{2}}}_{\text{prior}}}\text{+ }\frac{\text{1}}{{\text{σ}^{\text{2}}}_{\text{ev}}}}\text{ + }\frac{\frac{\text{1}}{{\text{σ}^{\text{2}}}_{\text{ev}}}\text{ }}{\frac{\text{1}}{{\text{σ}^{\text{2}}}_{\text{prior}}}\text{+ }\frac{\text{1}}{{\text{σ}^{\text{2}}}_{\text{ev}}}}\text{= }\frac{\frac{\text{1}}{{\text{σ}^{\text{2}}}_{\text{prior}}}\text{+ }\frac{\text{1}}{{\text{σ}^{\text{2}}}_{\text{ev}}}\text{ }}{\frac{\text{1}}{{\text{σ}^{\text{2}}}_{\text{prior}}}\text{+ }\frac{\text{1}}{{\text{σ}^{\text{2}}}_{\text{ev}}}}\text{ =}\text{ }\text{1}$ (2.4)

And so:

$\text{w}_{\text{prior}}\text{=}\text{1}\text{-}{\text{ }\text{w}}_{\text{ev}}$ (2.5)

Note that Eqs 2.1 and 2.5 are identical to Eqs 1.1 and 1.2 which describe our model.

This means that it is possible to interpret the parameter $\text{w}_{\text{prior}}$ in our model in Bayesian terms, as an agent’s estimate of the precision (or confidence) of their expectations relative to the precision of the incoming evidence provided by the senses. Thus, if expectation and evidence are judged to be equally reliable $\text{w}_{\text{prior}}$ = .5. In contrast, values of $\text{w}_{\text{prior}}$<.5 suggest that agents believe the incoming evidence is more reliable than prior beliefs (and vice versa if $\text{w}_{\text{prior}}$ >.5).

However, while this equivalence between Bayesian inference and our model can be shown mathematically, in the present work we do not directly measure or manipulate ${\sigma^{2}}_{ev}$ or ${\sigma^{2}}_{prior}$. As such, any value of $\text{w}_{\text{prior}}$ used by an agent in our point-estimate model should be thought of as an *as if* Bayes-optimal inference (e.g., a participant whose behaviour is best fit by a value of $\text{w}_{\text{prior}}$ = .5 is behaving in a Bayes-optimal fashion, if *they believe* incoming evidence and their prior beliefs are equally reliable, but we cannot verify whether this is true).

Future work directly measuring or manipulating variables implied by ${\text{σ}^{\text{2}}}_{\text{ev}}$ or ${\text{σ}^{\text{2}}}_{\text{prior}}$ (e.g., manipulating the uncertainty in evidence or expectations) will be important for determining whether we should conceptualise this kind of learning in fully Bayesian terms, rather than the simpler mechanics of the point estimate model described in Eqs 1.1 and 1.2.

1. **Quantifying model fit**

We primarily evaluated our model in the main manuscript by evaluating how closely effects simulated by the model corresponded to the empirical bias displayed by each participant. This revealed a tight correlation – *r =* .731, *p*<.001. Capturing these effects is the primary metric we use to evaluate the model, and indeed the mean squared error associated with these predictions is MSE = .0007.

We ran additional analyses to verify how well the visibility ratings and inferred beliefs simulated in our model matched the empirical visibility ratings made by each participant. We assessed whether visibility ratings simulated trial-by-trial for each participant correlated with their empirical visibility ratings – yielding a correlation for each subject. Across participants, the correlations are positive (mean *r* = 0.237, *t* = 16.541, *p < .*001, MSE = 0.044). This suggests that the computations implied by the model can capture individual variability in visibility ratings – and the average mean squared error was MSE = .044.

In a second approach to answering this question we evaluated whether the inferred belief trajectory in the model (rather than the simulated ratings) correlated with true visibility ratings across trials. This also found positive correlations across subjects (mean *r* = 0.233, *t =* 15.331*, p*<.001). The average mean squared error was MSE = 0.155. However, this second result may be unsurprising, given that simulated visibility ratings used in the first analysis are simply a linear transformation of the signal strength inference used in the second.

1. **Comparison of computational models with and without transfer function**

Our model assumes that agents generate subjective visibility ratings by forming an inference about signal strength on a given trial and passing this inference through a logistic transfer function. This model architecture mirrors the hypothetical cognitive architecture we assume agents actually use – with agents forming beliefs about signal strength but communicating them in potentially noisy or biased ways. This is similar to recent ideas in metacognition research (Bang et al., 2020; Guggenmos, 2022) and is also consistent with ideas from reinforcement learning (Lockwood & Klein-Flügge, 2021), where models typically assume that decisions are made by passing internal beliefs (about value) through a noisy transfer function that generates the overt choice (NB: in these models this noise parameter is often called the ‘inverse temperature’).

However, an alternative possibility is that agents do not ‘transform’ their beliefs about signal strength to produce a visibility rating but make these ratings by reading out these inferences directly. If this is the case, a simpler model – without a transfer function – would provide a better fit to our data.

We evaluated this possibility by fitting and simulating data from a simpler model without a transfer function and comparing this to the model in the main text. Like our main model, this simpler model could also reproduce the same bias in visibility ratings on Expect Strong and Expect Weak trials – and there was a significant correlation between the empirical effects seen for each participant and those predicted by the model – r = .520, p<.001.

While this suggests a simpler model without a transfer function can account for the qualitative patterns in our data, the fit of this simpler model to the real empirical data (r squared = .270) is substantially poorer than that of our main model including the transfer function (r squared = .534). Indeed, comparison of adjusted r square values – which correct model fit for the number of free parameters in each model – suggests that the fit is substantially better for our main model (adjusted r squared = .510) than for simpler model without a transfer function (adjusted r squared = .258). This justifies the use of this slightly more complex model, over a simple model where inferences of signal strength are read out directly.

***Supplementary references***

Bang, D., Ershadmanesh, S., Nili, H., & Fleming, S. M. (2020). Private–public mappings in human prefrontal cortex. *eLife*, *9*(e56477).

Dayan, P., & Kakade, S. (2000). Explaining Away in Weight Space. *Advances in Neural Information Processing Systems*, *13*.

Guggenmos, M. (2022). Reverse engineering of metacognition. *eLife*, *11*(e75420).

Lockwood, P. L., & Klein-Flügge, M. C. (2021). Computational modelling of social cognition and behaviour—A reinforcement learning primer. *Social Cognitive and Affective Neuroscience*, *16*(8), 761–771.

Rescorla, R. A., & Wagner, A. R. (1972). A theory of Pavlovian conditioning: Variations in the effectiveness of reinforcement and nonreinforcement. *Current Research and Theory*, 64–99.
